# Harnessing artificial intelligence for enhanced veterinary diagnostics: A look to quality assurance, Part II External validation

**DOI:** 10.1111/vcp.13407

**Published:** 2025-01-22

**Authors:** Christina Pacholec, Bente Flatland, Hehuang Xie, Kurt Zimmerman

**Affiliations:** ^1^ Department of Biomedical Sciences and Pathobiology Virginia‐Maryland College of Veterinary Medicine Virginia USA; ^2^ Department of Biomedical and Diagnostic Sciences University of Tennessee Institute of Agriculture Knoxville Tennessee USA

## Abstract

Artificial intelligence (AI) is emerging as a valuable diagnostic tool in veterinary medicine, offering affordable and accessible tests that can match or even exceed the performance of medical professionals in similar tasks. Despite the promising outcomes of using AI systems (AIS) as highly accurate diagnostic tools, the field of quality assurance in AIS is still in its early stages. Our Part I manuscript focused on the development and technical validation of an AIS. In Part II, we explore the next step in development: external validation (i.e., in silico testing). This phase is a critical quality assurance component for any AIS intended for medical use, ensuring that high‐quality diagnostics remain the standard in veterinary medicine. The quality assurance process for evaluating an AIS involves rigorous: (1) investigation of sources of bias, (2) application of calibration methods and prediction of uncertainty, (3) implementation of safety monitoring systems, and (4) assessment of repeatability and robustness. Testing with unseen data is an essential part of in silico testing, as it ensures the accuracy and precision of the AIS output.

## 1 INTRODUCTION

Our Part I manuscript discussed model development or technical validation of artificial intelligence systems (AIS) for medical use. Considerations, such as the intended purpose, computational and hardware requirements, data set considerations, expectations for interpretability and explainability, metric selection and decision rules, and model selection and performance evaluation, were discussed.

**FIGURE 1:**
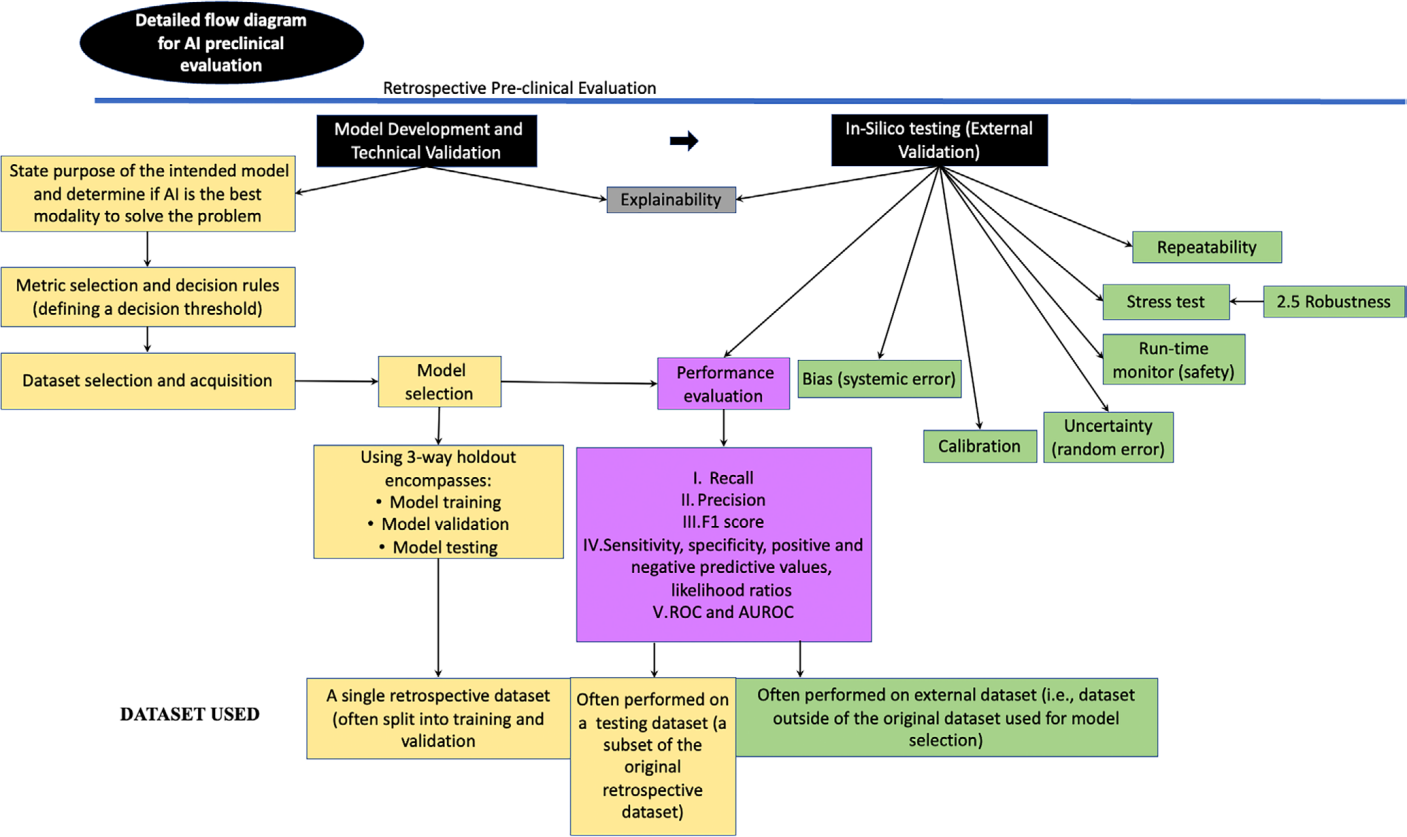
A comprehensive analysis of the requirements for preclinical evaluation of an artificial intelligence system (AIS). Adapted from the FUTURE‐AI and DECIDE‐AI guidelines. (Lekadir K, Feragen A, Fofanah AJ, et al. FUTURE‐AI: International consensus guideline for trustworthy and deployable artificial intelligence in healthcare. 2023. arXiv, 230912325; Vasey B, Novak A, Ather S, Ibrahim M, McCulloch P. DECIDE‐AI: a new reporting guideline and its relevance to artificial intelligence studies in radiology. Clin Radiol. 2023;78(2):130‐136.) Part I of this review covers model development and technical validation, and Part II covers in silico testing (external validation). The bottom of the figure highlights the need for separate data sets to be used in model development and in silico testing stages of development. Performance evaluation is used in all stages of development and deployment of an AIS. Additionally, if explainability is required for the model, then this should be addressed in both model development and in silico testing (external validation) phases of development. Model development and in silico testing are performed preclinically (outside of a clinical setting) and retrospectively, whereas subsequent phases of AI development, including shadow‐mode evaluation, early‐stage live evaluation, comparative trials, and monitoring, are performed within the clinic (prospectively).^1^, ^2^

The next step in AIS development for medical use is external validation or in silico testing. Readers should be aware that these are considered synonyms throughout the literature on AI. In this work, we discuss the intended purpose, components, and importance of in silico testing before shadow‐mode evaluation (offline validation), where the AIS analyzes real‐time data, but its output remains in the laboratory. As outlined in Figure 1, both model development and in silico testing phases of development are performed preclinically and with cases collected retrospectively, whereas the subsequent phases of development are performed within the clinic and with prospectively enrolled cases.

## 2 IN SILICO TESTING

In silico testing can be described as a virtual clinical trial, where the AIS will undergo more rigorous testing on a new data set. The new data set should represent the patient population for which the AIS is intended but be collected separately (e.g., at a different time point) from the data set used in the model development phase. FUTURE‐AI and related literature recommend the following core topics be evaluated for any AIS intended for use in health care (Figure 1): (1) bias, (2) calibration, (3) uncertainty, (4) run‐time monitoring, (5) generalizability, (6) robustness, (7) repeatability, (8) stress test, and **±** explainability. The current recommendation is that if in silico testing is to be performed, this should be considered early in AIS development to facilitate overlap between model development and in silico testing phases, where appropriate.^3^

### 2.1 Bias

In veterinary clinical pathology, the bias (inaccuracy) of a quantitative test is represented by the difference between the measured results and a known standard or method (representation of “true” analyte concentration). Sources of bias in AIS development are more complex and have been categorized as (1) human versus machine, (2) based on the stage of AIS development in which bias is introduced, or (3) statistical (arising from the model or algorithm such as unbalanced data classes) versus social (i.e., human bias such as stereotypes).^4^, ^5^ In this paper, we will use the categorization of bias set forth by the National Institute of Standards and Technology (NIST), which divides bias into three major categories: statistical, human, and systemic.^6^

*Statistical bias* is defined similar to bias (inaccuracy) in analytical, quantitative method validation as “a systemic tendency for estimates or measurements to be above or below their true values.”^7^ In AIS model development, statistical bias can originate from different sources, including the data or the algorithmic processes used during AIS development. Statistical bias can be introduced during (1) data acquisition, cleaning, or processing and (2) model development (training, validation, testing, and selection).^6^ Statistical bias during training, validation, and testing can include optimistic bias (overestimating true accuracy), pessimistic bias (underrepresentation of the true accuracy), and error propagation (when an AIS generates a biased output that is then used as an input for other AIS), to name a few.^6^, ^8^ Statistical bias may not only have legal, ethical, and fairness concerns but also affect the accuracy and generalizability of a model.^9^ When statistical bias occurs, an AIS performs well on training data but poorly when unseen data sets or clinical data are used.

*Human bias* arises from applying overly simplistic “rules of thumb,” which are used to alleviate the requirement for deep thinking about a process to arrive at a conclusion. These types of biases include *automation complacency*, when a human's reliance on an AIS becomes too great, often leading to degraded skills over time by overuse of an AIS for decision‐making; *automation bias*, when a human shows excessive trust in the decision‐support system despite contradicting evidence such as physical exam findings, bloodwork changes or history; *anchoring bias*, when a person relies on one, and often the first, piece of information to draw a conclusion; and *confirmation bias*, when a person interprets new information as supportive of a preexisting belief or expectation.^6^, ^10^ These biases are fundamental to human nature because they permit people to more quickly process information, reducing the complexity of a task or decision. However, these human “interpretive shortcuts” can influence all stages of AIS development and quality assurance, and steps must be taken to prevent or mitigate their effects.

*Systemic bias* occurs when inequities in the AIS lead to poorer outcomes for a given group of patients, often an underrepresented group.^6^, ^11^ *Systemic biases*, including historical, institutional, or societal biases based on gender, race, and age, are easily introduced into the AIS. These biases codify discrimination and decrease diagnostic accuracy, leading to poorer outcomes, harm in a given group of patients, and even propagate real‐world discrimination.^6^

Bias in AIS development, when evaluated, is often not investigated until after the model selection process; however, bias should be evaluated throughout each stage of AIS development, especially prior to data collection and algorithm selection.^5^ In analytical, quantitative method validation, inaccuracy is assessed using either method comparison or recovery experiments as part of initial method validation.^12^

An example to demonstrate how bias could negatively impact veterinary patients is if an AIS is designed to detect not lymphoma and lymphoma from cytology images and information on the breed of the dog. Suppose 90% of all golden retriever cytology images are used to train and validate the AIS. When the AIS is used in a clinic and given a cytology image of a golden retriever lymph node, the system may be biased in diagnosing lymphoma based on the breed information, potentially harming patients without lymphoma.

In AISs, ways to assess bias include data analysis tools, bias monitoring systems, fairness metrics, and computer simulations.^6^, ^13^, ^14^ Another tool on the horizon for bias identification and mitigation in AI is the Prediction Model Risk of Bias Assessment Tool Early‐AI.^15^ This program intends to guide AIS developers through risk mitigation in diagnostic and prognostic AIS, likely using a questionnaire‐based format. However, more research into understanding the best ways to detect, categorize, and mitigate bias during AIS development is needed to reduce patient harm.

### 2.2 Calibration

The next step in the in silico testing of an AIS is calibration. Adequate calibration allows clinicians to make medical interpretations based on probabilities. Calibration of today's more complex AIS output is critical for trustworthiness and correct clinical interpretation.^16^ In the clinical setting, an ideal AIS would provide the original uncalibrated output alongside a calibrated confidence level, that is, the probability that the assigned “class label,” or predicted category assignment, is accurate.^16^

Calibration is performed by considering the model's diagnostic accuracy and the output value for a specific image. This allows the output classification to be interpreted as a diagnostic probability, as described above. Calibration is an ongoing area of research, and best practices have not yet been established. For current recommendations on detailed calibration methods, see Maier‐Hein et al. *Metrics Reloaded: Recommendations for image analysis validation*.^17^

For an example, let us return to the hypothetical AIS that can predict large‐cell lymphoma or not lymphoma in canine patients. In this scenario, the AIS outputs a continuous numerical value on a continuous scale between 1 and 0. An output of 0 represents no lymphoma, and an output of 1 represents large‐cell lymphoma. If an image is put into the AIS and the output is 0.8, one might interpret this result as a probability (e.g., there is an 80% chance the image is large‐cell lymphoma). However, if the AIS output is not calibrated to clinical outcomes, this interpretation is inappropriate, and a clinician does not know how to appropriately interpret a value of 0.8 (i.e., the clinician cannot know how confident the model is that the image is large‐cell lymphoma). This lack of calibration could lead to misinterpretation and potential patient harm. If the model is calibrated to clinical outcomes, it may be possible to interpret the output of 0.8 as an 80% chance that the patient's image represents large‐cell lymphoma.

### 2.3 Uncertainty

Uncertainty in an AIS is the lack of trust in the model's output (predictions).^18^ Measuring the uncertainty of an AIS can help clinicians understand what conclusions should and should not be drawn from the model output for a given patient. In veterinary clinical pathology, sources of measurement uncertainty include preanalytical factors, analytical variation, and biological variation. The importance of measurement uncertainty to results interpretation is well established; many clinicians do this informally and intuitively. Veterinary diagnostic laboratories in the United States do not routinely report measurement uncertainty with patient laboratory data. Expanded measurement uncertainty (EMU) and dispersion (D) are ways of communicating uncertainty in individual patient results from quantitative measurement methods.^19^

AISs have been commonly considered “black boxes,” meaning often‐users do not understand how the model is making its predictions and accept results at face value. Researchers have pushed for explainable AI (XAI) models to improve critical interpretation of AIS output to reduce uncertainty and show an AIS's trustworthiness. However, even when AIS output is explained, output may not be accurate. AIS, which also expresses output uncertainty, can help clinicians understand when to rely on the model and when to use caution when drawing conclusions from the output.^20^, ^21^ Expressing the uncertainty of a model's output is critical in AI intended for high‐risk settings (i.e., where the incorrect output can have serious consequences). Veterinary medicine is considered a high‐risk setting, where a wrong output could lead to inappropriate diagnosis, delayed treatment, or even euthanasia of a patient.

AIS developers refer to two general categories of uncertainty: *aleatoric* and *epistemic*. Aleatory, or data uncertainty, is inherent randomness and is often described as unreducible for a data set. It results from information loss inherent to the data, such as noise in the data, image resolution, or data classification overlap.^22^ If data quality can be improved (e.g., through resolution enhancement), one might achieve reduced uncertainty.^23^ Epistemic, or model uncertainty, refers to the uncertainty associated with the AIS model. It is a measure of how well‐model parameters match the data.^22^ For example, causes of epistemic uncertainty include a lack of data to cover all aspects of the intended AIS use, errors in the model training procedure or poor model structure (e.g., hyperparameter selection).^20^ One method of reducing epistemic uncertainty in a model is by showing the model more data during training and validation.^20^

Distributional uncertainty is a third type of uncertainty in AIS. It refers to the uncertainty in a prediction output that arises when there is a change in the input data distribution.^20^ In other words, this uncertainty occurs when the training data used to make the AIS does not cover a region of input data the AIS will encounter in the testing data set. Distributional uncertainty occurs commonly in real‐world problems due to the variability inherent to most clinical environments (e.g., variability in personnel, hardware, software, and samples).^20^ This type of error can be reduced by adding data to the training data set that covers the previously underrepresented data region.^20^ This is different from epistemic uncertainty, which occurs during the building and training of the AIS.^20^

While having AIS report uncertainty around their outputs is desirable, it is worth noting that some AIS, which include uncertainty prediction, have high computational and memory requirements. Therefore, uncertainty computing and reporting may not always be possible in real‐world clinical applications.^20^

In addition to high computational and memory requirements, other limitations of computing and reporting uncertainty in real‐world clinical settings include the inability to provide uncertainty for a single output prediction (uncertainty output is based on an entire testing data set), the lack of ability to validate expressions of uncertainty, the lack of standardized protocols to implement uncertainty measurements, the lack of structured evaluation of currently available uncertainty quantification methods, and the inexplicability of uncertainty outputs.^20^ Many unanswered questions still require attention from the AI research community before expressions of uncertainty can be routinely implemented in a clinical setting. Establishing a protocol that indicates the uncertainty surrounding a single instance (single patient input) would help increase the reliability, interpretability, and trust clinicians have for AIS since clinicians most commonly make decisions for individual patients rather than patient populations.^20^

### 2.4 Run‐time monitors

Run‐time monitors are employed in AIS to identify unsafe predictions and remove them before they lead to a negative consequence (i.e., the clinician interprets the output as truth, leading to a potentially negative impact for the patient). Consider these models as an extra piece of software trained alongside the AIS to improve AIS safety in high‐risk settings (i.e., when an incorrect output prediction/diagnosis given by an AIS leads to severe consequences such as loss of life). Generally, AIS intended for use in the medical field or automated driving applications are considered high‐risk settings. There are currently two different perspectives on the best way to monitor safety. The first is out‐of‐distribution (OOD), which detects input data outside the training data set distribution. Most run‐time monitoring has focused on the OOD approach, which has limitations.^24^ OOD detection methods (1) sometimes discard valid predictions or accept wrong predictions, (2) are difficult to compare, meaning it is challenging to select which OOD is best for a given AIS, and (3) the concept of OOD is poorly defined and requires implementation of a threshold at which the output is discarded, which must be defined. For these reasons, Guerin et al. propose using out‐of‐model scope (OMS) detection instead. OMS focuses on predictions rather than data distributions and detects input data that results in incorrect predictions.^24^

For example, suppose an OOD is produced for an AIS that detects lymphoma or not lymphoma in canine cytology samples. In that case, the AIS will be trained on the training dataset, and during this process, the OOD monitor will also be trained to identify safe data instances from unsafe ones. When the AIS is put into a clinic and fed a new image, the OOD will compare the new image to the safe data instances it learned from the training dataset. If the new image is different, the prediction will be rejected; if the new image is not different from the training dataset (i.e., pixel number, resolution, and brightness are similar to training images), the prediction may be accepted. However, what happens if the OOD classifies a new image as safe data because it is similar to the training data, but the output prediction from the image should be considered unsafe (because of some uncertainty associated with the prediction)? This is where OMS is used because OMS focuses on the prediction (i.e., the uncertainty surrounding the prediction), not on the input data. So, in this scenario, the OMS may still detect the poor prediction, even when OOD would have allowed the prediction with high confidence to be made.

To evaluate if a run‐time monitor is functioning as intended (removing unsafe predictions), Guerin et al. propose the use of three metrics: (1) safety improvements with the run‐time monitor, measured by recall (sensitivity), (2) remaining hazards in the system, measured by false negative rate (1‐sensitivity), and (3) system performance decrease due to the safety monitor implementation, measured by false positive rate (1‐specificity).^25^ The impact of the monitoring system on the AIS can be evaluated by comparing recall, false negative rate, and false positive rate before and after implementation of the safety monitor. These metrics are commonly reported in the output of a model, so it is convenient if these same values can be used to validate a run‐time monitoring system.^25^

### 2.5 Robustness

Robustness describes the ability of an AIS to function correctly when invalid inputs or stressful environmental conditions (e.g., differences in equipment) are encountered.^1^, ^26^ There are two main branches of robustness: (1) natural and (2) adversarial robustness.

Natural robustness (nonadversarial robustness) is the ability of an AIS to maintain performance when it encounters naturally induced image corruptions or perturbations that cause differences in the training and testing data.^27^ When achieved, natural robustness would help a model perform well when distributional shifts in the data are present, that is, a mismatch between the data used to train the AIS and the data from the intended environment. This is especially desirable because distributional shifts occur when AISs are implemented in different hospital environments.^27^, ^28^ An example of this is a model trained to detect lymphoma from not lymphoma in canine patients using cytology samples collected from a referral center with intended deployment in rural settings. The AIS may perform worse in rural settings due to differences in disease prevalence, hardware and software, personnel, and image capture technologies.

Adversarial robustness describes the ability of a model to maintain its performance in the face of adversarial attacks or nonrandom perturbations.^28^ In the case of image analysis, perturbations are imperceptible modifications to the input (i.e., changes that cannot be detected by visual inspection of the image) that change the model's prediction. Adversarial attacks can be targeted or untargeted. When targeted, the attack causes an input to be intentionally misclassified into a specific incorrect class. When untargeted, the attack causes an input to be misclassified in general. Regardless, when present, these issues maximize a model's error.^28^

Training using large data sets that adequately represent the intended patient population is one of the best ways of ensuring a robust AIS. There are many different approaches to achieve more robust models, such as (1) intentionally modifying input data in the training data set to represent the variability (noise) that can be encountered in real‐world environments, (2) pushing the model to learn more robust features, that is, a model capable of maintaining high output accuracy in the face of changes in microscopes or cameras used to capture the input images, or (3) modifying the AIS to mitigate the effect of noise on the output, to name a few.^28^ Given the number of resources that go into creating an AIS, its generalizability across different hospital environments and when shifts in data are encountered becomes an essential feature for success.

### 2.6 Repeatability

Repeatability is at the core of all sound scientific experiments, and in AIS, it is closely related to the trustworthiness of a system. Repeatability is, therefore, a requirement for successful AIS implementation in hospitals. Unfortunately, the lack of repeatability in AI research remains a limitation of its use. The primary reasons for the lack of repeatability are (1) the inherent way AI algorithms are constructed and (2) the lack of uniformity by researchers to report all necessary information to allow assessment of repeatability.^29^ A ‘weight initialization’ process is performed when an AIS is constructed. The weights (an input feature's impact on the output) are initially determined using an internal randomization process. Because the weight value is determined randomly, even the same research team cannot reproduce the same results. Furthermore, even when weights are initiated the same way (nonrandomly), reproducibility is still elusive due to variations in hardware and software versions. Alahmari et al.^29^ achieved reproducible results by fixing all randomizations, such as weight initialization. However, even when reproducibility can be achieved by the originating researcher, Gundersen and Kjensmo found in 2018 that only 20%–30% of the variables required to reproduce results are given in empirical AI research and that none of the papers evaluated in their study provided all the information needed to replicate results.^30^

Recently, in recognition of this problem and others like it, several consensus statements have been released or are in production to standardize reporting and support reproducibility in AI research. These consensus statements are relative to the stage of development of the research. For example, TRIPOD‐AI and STARD‐AI are intended for offline validation, whereas SPIRIT‐AI and CONSORT‐AI are intended for clinical trial settings. An international consensus, FUTURE‐AI covers all stages of development from design to deployment and monitoring. Regarding reproducibility, FUTURE‐AI states that all AI tools should undergo repeatability testing.^1^ Documenting repeatability is essential before deploying these systems in the medical field.

### 2.7 Stress testing for generalizability

**FIGURE 2:**
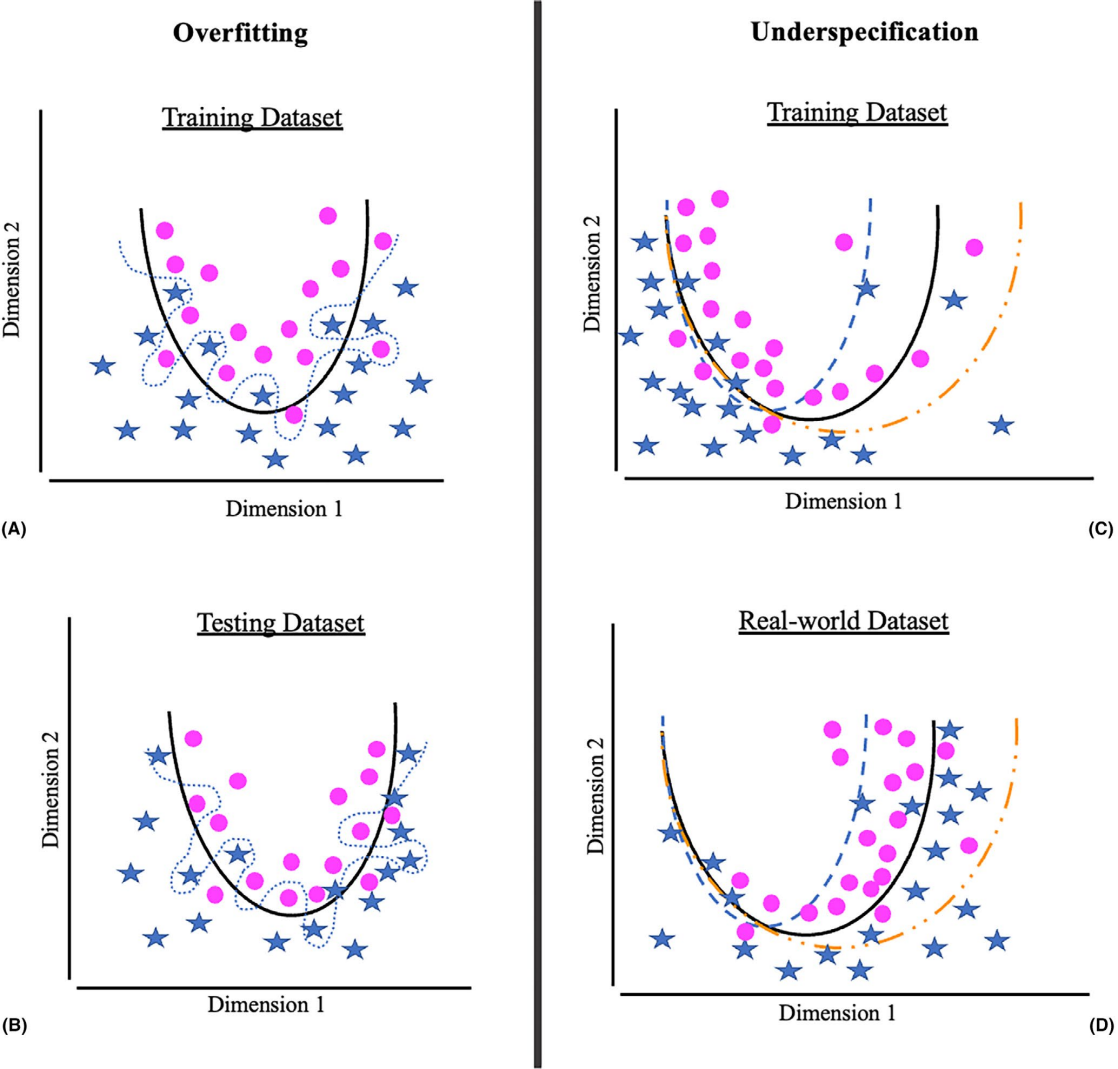
Graphical representation of overfitting and underspecification. Adapted from Figure 1 in Eche T, Schwartz LH, Mokrane F‐Z, Dercle L., Toward Generalizability in the Deployment of Artificial Intelligence in Radiology: Role of Computation Stress Testing to Overcome Underspecification, Radiology: Artificial Intelligence, 2021 3:6.. The blue stars and pink dots represent different shapes an AI is trying to correctly categorize. The dimension references the space the AI is using to partition the two shapes, in this case, stars and round dots. A and B represent overfitting, and C and D represent underspecification. (A) The solid black line represents the ideal model (i.e., the model that will best perform the provided task), and the dotted blue line represents an overfitted model. The model is “overtrained” or has memorized the training data set rather than finding true predictive features that help it classify round versus star shapes. This model will have a high accuracy on the training data set. (B) When the model is presented with the unseen testing data set, it fails to successfully distinguish between round and star shapes. The accuracy of this model will be low. (C) Three models are trained using a training data set and are represented as Model 1, dotted blue line; Model 2, solid black line; and Model 3, dotted yellow line. All three models have a similar performance on the training data set. (D) Three models are evaluated on an unseen, real‐world data set that has a distribution shifted to the right along dimension 1. When the models are evaluated with the real‐world data set, only Model 2 (solid black line) will maintain a high performance. This is the model that should be used.^32^

Stress testing assesses the generalizability and robustness of an AIS and evaluates a model's readiness to be tested in a clinical setting.^1^, ^31^ There is a lack of consensus on what processes encompass stress testing, but some describe it as detecting and minimizing underspecification.^32^ Underspecification occurs when different models perform well on a training data set, but when real‐world data are used, they perform poorly. Figure 2 compares the differences between overfitting and underspecification.^33^ Underspecification is one reason AIS commonly fails when placed into real‐world situations.^31^

A key feature of underspecification is that it cannot be identified during the AIS validation phase because the testing data set has an identical disease distribution to the training and validation data sets.^32^ Underspecification can be confused with another common problem: overfitting. Overfitting occurs when the AI model cannot distinguish noise detected during training from authentic predictive features.^32^ This happens when the model is overtrained to the training data, picking up on features that are not genuinely predictive (i.e., an overtrained model has “memorized” the training data, but when shown the unseen testing data, it will perform poorly). Overfitting differs from underspecification in that it can be detected during the testing phase of method validation.^34^

Three ways to perform stress testing include (1) shifted performance evaluation (i.e., shift the data set by changing the resolution of the images or changing the staining protocol of the pathology slides) and see if overall performance changes, (2) contrast evaluation (i.e., shift the data set but instead of evaluating overall performance, look at each instance/prediction of each image, and determine if changes have occurred), and (3) stratified performance evaluation (i.e., break the data set into subgroups such based on which camera was used to collect the images and evaluate performance).^32^, ^33^ Overall, the best methods for stress testing need to be assessed and implemented before a model is used in a clinical setting. This is an area of research that is still being explored.

## 3 CONCLUSION

Despite the recent successes of AIS in research settings where they perform as well as or better than medical professionals on a given task, many unanswered quality assurance questions remain. Reporting performance metrics alone may be acceptable proof of concept during model development. However, in silico testing requires rigorous testing to ensure the output quality before a system enters a clinical setting to minimize the risk of patient harm.

Evaluation of an AIS during in silico testing should include evaluation for bias, calibration, uncertainty, implementation of run‐time monitors (safety), system robustness, repeatability studies, and stress testing for generalizability. Despite knowing that an AIS should be interrogated for these qualities, the best way to do so, in many cases, remains unknown.

As this field develops, guidelines that standardize AIS quality assurance will be helpful to maintain high standards of diagnostics in veterinary medicine. Additionally, guidelines like FUTURE‐AI and SPIRIT‐AI will help standardize AIS reporting and allow for transparency that will support trust and advance the field forward.

Following technical validation and in‐silico, there are subsequent development phases beyond the scope of this paper. However, they include shadow‐mode evaluation (offline validation), early‐stage live evaluation, comparative trials, and monitoring. Each of these phases strengthens the trust, ensures quality, and determines the clinical efficacy of an AIS as it is implemented in its intended environment.

Looking to the future, the prospects of AI in veterinary clinical pathology are vast. We anticipate further advancements in machine learning algorithms to provide more accurate and nuanced insights into animal health. There is potential for developing AIS that are more adaptable and capable of learning from a wider variety of data sources, leading to more comprehensive and holistic approaches to animal health care. Successful implementation of AIS in all fields of veterinary medicine requires a collaborative approach between all stakeholders from the beginning to ensure that quality diagnostic testing continues to be the standard in veterinary medicine.

## CONFLICT OF INTEREST STATEMENT

The authors declare no conflicts of interest.

## APPENDIX A

1. **Algorithm:** A set of rules or instructions given to an AI program to help it learn and make decisions.
2. **Artificial Intelligence (AI):** Computers performing tasks that usually require human intelligence, like recognizing patterns or solving problems.
3. **Artificial Intelligence Model:** A system that recognizes specific patterns from a provided data set.
4. **Bias:** In AI, bias refers to an algorithm's tendency to favor certain outcomes based on its design or the data it is trained on.
5. **Calibration:** Adjusting AI predictions to make them more accurate and reliable in real‐world applications.
6. **Data Acquisition:** The process of collecting data to train and test AI systems.
7. **External Data set:** A data set derived from a different source than the internal data set.
8. **External Validation:** Evaluation of a model using a data set that was collected separately from the training (internal) data set.
9. **Explainability:** The ability of an AI system to clearly explain how it arrived at its decisions or predictions.
10. **Fairness:** Ensuring that AI systems operate without bias, providing equitable outcomes across all groups regardless of attributes like race, gender, or age.
11. **Generalizability:** The ability of an AI system to apply what it has learned from specific data sets to new, unseen data.
12. **Hyperparameter:** A set of parameters the researcher selects before the model is trained.
13. **In Silico Testing:** Testing AI models using computer simulations instead of real‐world experiments.
14. **Internal Data set:** The internal data set is often derived from information collected from within a specific organization (e.g., hospital) and is frequently used to train an AI model.
15. **Model:** A program that detects a specific pattern learned from training data.
16. **Model Development:** Creating and designing an AI model includes selecting the right approach and setting performance goals.
17. **Overfitting:** When a model performs well on the training data set but poorly on a new, unseen data set.
18. **Parameter:** Distinguishing features or variables, the AI learns during training and then uses to make its predictions.
19. **Robustness:** The strength of an AI system is its ability to maintain performance even when faced with challenging or unexpected conditions.
20. **Run‐time Monitoring:** The process of continuously checking an AI system as it operates to ensure it is working correctly and safely.
21. **Stress Test:** Any test used to evaluate the stability and robustness of a model.
22. **Underfitting:** When a model performs poorly on the training data set and new, unseen data set because it cannot capture the relationship between the input and output.
23. **Underspecification:** When several different models perform well on the training data set, but when deployed, their performance decreases due to differences in the training and deployment environments.
24. **Uncertainty:** The confidence level in an AI system's predictions. High uncertainty means the system is less sure about its predictions.
25. **Variance:** How changes in the data used to train an AI system can impact its performance.
